# When Coagulase-Negative Staphylococci Mislead: Transient Metabolic Acidosis in a Healthy Toddler

**DOI:** 10.7759/cureus.92689

**Published:** 2025-09-19

**Authors:** Filippos Filippatos, Dimitra-Ifigeneia Matara, Vasiliki Karava, Konstantinos Kakleas, Athanasios Michos

**Affiliations:** 1 First Department of Pediatrics, Medical School, National and Kapodistrian University of Athens, Athens, GRC; 2 Department of Nephrology, Agia Sofia Children's Hospital, Athens, GRC; 3 Department of Allergy and Immunology, Agia Sofia Children's Hospital, Athens, GRC; 4 Infectious Diseases and Chemotherapy Research Laboratory, Medical School, National and Kapodistrian University of Athens, Athens, GRC

**Keywords:** bloodstream infection, coagulase-negative staphylococci, lower respiratory tract infection, metabolic acidosis, pediatric, staphylococcus hominis

## Abstract

*Staphylococcus hominis *(*S. hominis*), a coagulase-negative staphylococcus (CoNS), is commonly encountered in blood cultures and presents diagnostic challenges in both pediatric and adult infections. Its clinical significance remains uncertain, especially in previously healthy children without evident immunodeficiency or indwelling devices. Metabolic acidosis associated with *S. hominis* bacteremia is rarely reported, further complicating clinical interpretation. We report a previously healthy two-year-old boy presenting with high-grade fever, cough, mild respiratory distress, and metabolic acidosis (pH 7.28, bicarbonate 12 mmol/L). Initial blood culture yielded *S. hominis*, while subsequent cultures remained negative. The patient had no immunodeficiency or indwelling devices. Clinical improvement occurred rapidly with supportive care, inhaled bronchodilators, intravenous fluids, short-course corticosteroids, and intravenous antibiotics based on antibiogram. Based on the above, the diagnosis was transient metabolic acidosis associated with viral/bacterial respiratory tract infection. Symptoms resolved within 48 hours, with complete normalization of laboratory parameters. The *S. hominis* isolate was evaluated based on clinical and microbiological reassessment, allowing antibiotic de-escalation and discharge on day four of admission. Differentiating true CoNS infection from contamination is critical in pediatric settings to prevent unnecessary antibiotic exposure. True *S. hominis* infections are exceedingly rare in immunocompetent children and typically associated with severe clinical manifestations and underlying risk factors. The transient metabolic acidosis observed likely resulted from mild dehydration and catabolic stress rather than direct bacterial effects. This case underscores the necessity for cautious interpretation of isolated CoNS-positive cultures, highlighting the importance of clinical correlation, repeated cultures, and careful risk assessment. The diagnostic dilemma presented by a single positive *S. hominis* blood culture in pediatric patients with transient metabolic acidosis emphasizes the need for judicious interpretation to avoid overtreatment. Further studies are warranted to better characterize the clinical significance of CoNS isolates in immunocompetent pediatric populations.

## Introduction

Coagulase-negative staphylococci (CoNS), such as *Staphylococcus hominis (S. hominis)*, are among the most common skin commensals and represent the leading cause of blood culture contamination in both adult and pediatric clinical practice [1,2]. In hospitalized children, mainly five days post-admission, CoNS bacteremia accounts for up to 40% of positive blood cultures, but distinguishing contamination from true infection remains a clinical challenge [2]. While *S. epidermidis *and* S. saprophyticus* are more frequently pathogenic, *S. hominis* has occasionally been implicated in invasive infections, mainly in immunocompromised hosts or those with indwelling devices [3,4].

Metabolic acidosis is an uncommon but potentially serious presentation in pediatric infections. It is most often linked to dehydration, catabolism, or sepsis but rarely to CoNS bacteremia [5]. The association of *S. hominis* with metabolic acidosis in otherwise healthy children is rarely described.

Here, we present a case of a young child with metabolic acidosis and a single *S. hominis*-positive blood culture and review the available literature to contextualize this finding and optimize the approach to diagnosis and management. This study was conducted in accordance with the Declaration of Helsinki. Institutional review board approval was not required for this single case report at our institution. Written informed consent was obtained from the patient’s parents for publication of this case report and any accompanying images.

## Case presentation

A previously healthy two-year-old boy was admitted to the pediatric department of a tertiary hospital with a 36-hour history of high-grade fever, peaking at 39°C, associated with a persistent cough without expectoration and mild respiratory distress. The child’s parents reported a significant reduction in oral intake over the preceding three days and noted a single episode of non-bloody, non-mucoid diarrhea. There was no known exposure to sick contacts, no recent travel, and no significant family or perinatal history of diseases/conditions.

**Figure 1:**
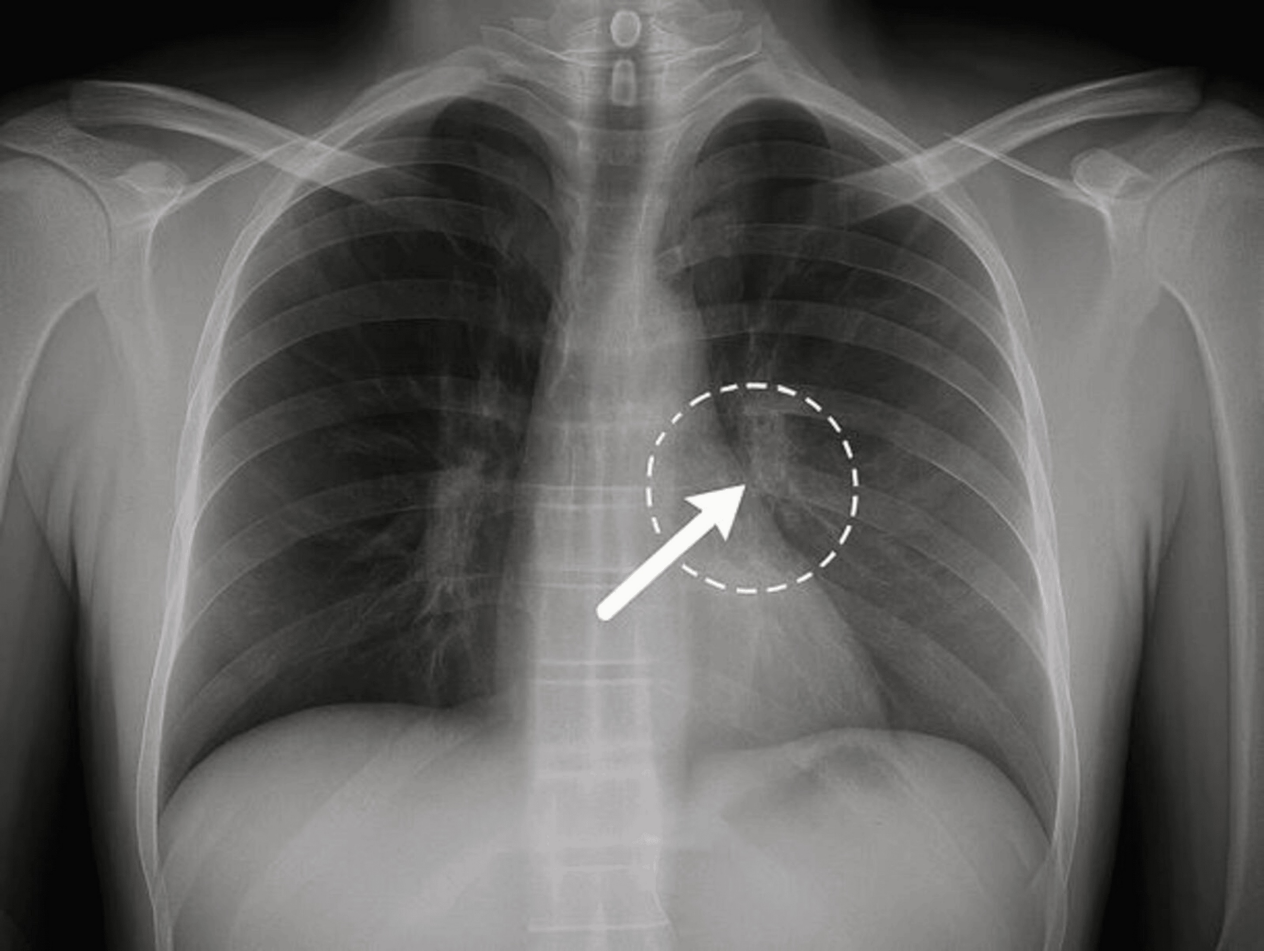
Chest radiograph demonstrating a subtle interstitial infiltrate in the left upper lobe (LUL), consistent with mild lower respiratory tract involvement. The arrow and dashed circle indicate a subtle interstitial infiltrate in the LUL. There was no evidence of consolidation, pleural effusion, or significant hyperinflation.

On initial examination, the child was alert and in a generally good condition. His weight was 10.9 kilograms, with a body temperature of 36.9°C (antipyretic was administered at admission to the Emergency Department), oxygen saturation of 98% on room air, heart rate of 126 beats per minute, and blood pressure measured at 104/77 mmHg (diastolic blood pressure: 95th percentile). Physical examination revealed mild periorbital hyperemia, erythematous tonsils, and mildly increased work of breathing characterized by expiratory wheeze and inspiratory crackles localized to the left upper lung field. Noted dehydration signs included reduced skin turgor, dry mucous membranes, and prolonged capillary refill time (3.5 sec). There was no evidence of lymphadenopathy or hepatosplenomegaly, and no other clinical dehydration signs were noted. The chest radiograph demonstrated a subtle interstitial infiltrate in the left upper lobe, consistent with mild lower respiratory tract involvement. No evidence of consolidation, pleural effusion, or significant hyperinflation was noted (Figure 1).

Laboratory investigations on admission showed a metabolic acidosis, with a blood gas revealing a pH of 7.28, pCO₂ of 26 mmHg, bicarbonate of 12 mmol/L, and lactate of 1.4 mmol/L. The complete blood count showed mild leukocytosis (WBC: 7.55x10³/μL) with a predominance of lymphocytes (47.8%), normal hemoglobin (12.0 g/dl) and platelet counts (265x10³/μL), and no features of hemolysis. C-reactive protein (CRP) and procalcitonin were mildly elevated at 3.25 mg/L (normal values: <5 mg/L) and 1.2 ng/mL (normal values: <0.5 ng/mL), respectively. Streptococcus pyogenes tonsilar infection was ruled out (rapid strep test and pharyngeal cultures were negative). Urinalysis was remarkable for glycosuria and significant ketonuria (+++), along with mild microscopic hematuria; urine culture was negative. The respiratory viral antigen panel was negative for respiratory syncytial virus, influenza, and adenovirus. Detailed laboratory findings at admission and discharge are summarized in Table 1.

**Table 1:** Laboratory findings at admission and discharge. CRP: C-reactive protein; SGOT: Serum glutamic‑oxaloacetic transaminase; SGPT: Serum glutamic‑pyruvic transaminase; γ-GT: Gamma‑glutamyl transferase; ALP: Alkaline phosphatase; LDH:  Lactate dehydrogenase; hpf: High‑power field

Parameter	Admission Value	Discharge Value	Reference Range	Units
Arterial Blood Gas				
pH	7.28	7.4	7.35–7.45	
pCO₂	26	23.2	35–45	mmHg
HCO₃⁻	12	14.6	22–28	mmol/L
Lactate	1.4	2.2	<2.2	mmol/L
Base Excess	–9.6	–7.9	–2 to +2	mmol/L
Inflammatory Markers				
CRP	3.25	<1.0	<5.0	mg/L
Procalcitonin	1.2	0.12	<0.5	ng/mL
Complete Blood Count				
WBC	7.55	6.8	5.0–14.5	x10³/μL
Neutrophils %	36.6	32	25–55	%
Lymphocytes %	47.8	48	35–65	%
Monocytes %	8.6	9	2–10	%
Hemoglobin	12	12.2	11.0–13.5	g/dL
Platelets	265	260	150–450	x10³/μL
Biochemistry				
Glucose	76	82	70–100	mg/dL
Urea	11	15	10–35	mg/dL
Creatinine	0.28	0.3	0.20–1.00	mg/dL
SGOT	46	35	10–60	U/L
SGPT	7	10	5–45	U/L
γ-GT	6	7	5–32	U/L
ALP	208	190	60–240	U/L
Total Bilirubin	0.25	0.2	<1.00	mg/dL
Albumin	4.8	4.9	3.7–5.5	g/dL
LDH	407	340	120–300	U/L
Magnesium	2	2.1	1.5–2.3	mg/dL
Potassium	4.1	4.2	3.5–5.5	mmol/L
Sodium	137	138	135–150	mmol/L
Chloride	103	104	95–110	mmol/L
Calcium	10.5	10.6	8.2–11.0	mg/dL
Urinalysis				
pH	5.5	6	5.0–7.5	
Specific gravity	1026	1018	1005–1030	
Protein	Negative	Negative	Negative	
Glucose	Positive	Negative	Negative	
Ketones	+++	Negative	Negative	
Blood	Positive	Negative	Negative	
WBC (urine, per hpf)	1–2	0–1	0–5	/hpf
RBC (urine, per hpf)	2–3	0–1	0–5	/hpf
Nitrites	Negative	Negative	Negative	
Bacteria	Few Gram (+)	None	None	

On the day of admission (hour 0), one aerobic blood culture was obtained, which yielded *Staphylococcus hominis* after incubation. Two subsequent blood cultures were collected at +24 hours and +48 hours post-admission, both of which showed no growth. Antibiogram with minimum inhibitory concentration (MIC) values is presented in Table 2.

**Table 2:** Antibiogram of Staphylococcus hominis isolate with MIC values and interpretation. Antibiotic susceptibilities were interpreted according to EUCAST/CLSI breakpoints. MIC: Minimum inhibitory concentration

Antibiotic	Interpretation	MIC (mg/L)
Penicillin	Resistant	≥0.5
Oxacillin (Methicillin)	Sensitive	≤0.25
Vancomycin	Sensitive	≤1.0
Teicoplanin	Sensitive	≤2.0
Linezolid	Sensitive	≤2.0
Daptomycin	Sensitive	≤1.0
Gentamicin	Sensitive	≤1.0
Ciprofloxacin	Sensitive	≤0.5
Clindamycin	Sensitive	≤0.25
Erythromycin	Resistant	≥8.0
Trimethoprim/Sulfamethoxazole	Sensitive	≤2/38
Rifampicin	Sensitive	≤0.5
Fusidic acid	Sensitive	≤1.0
Moxifloxacin	Sensitive	≤0.25

The patient received intravenous cefotaxime (150 mg/kg/day in three divided doses) and clindamycin (40 mg/kg/day in four divided doses), intravenous fluids, inhaled bronchodilators, and a three-day course of intravenous corticosteroids (methylprednisolone 1 mg/kg/day) for presumed lower respiratory tract infection. Cefotaxime was initially administered as empiric antibiotic coverage until blood cultures and antibiogram results were obtained. Within 48 hours, fever and metabolic acidosis resolved, and laboratory parameters normalized. Given the absence of persistent symptoms and negative follow-up cultures, the *S. hominis* isolate was deemed a contaminant after infectious diseases consultation. Antibiotics were de-escalated to oral amoxicillin/clavulanic acid (50 mg/kg/day of amoxicillin component in two divided doses). The patient received intravenous cefotaxime and clindamycin for two days, followed by oral amoxicillin/clavulanic acid, completing a total antibiotic course of seven days. The patient was discharged on day four in excellent condition with full clinical and laboratory recovery.

## Discussion

*S. hominis* is commonly encountered in blood cultures and presents diagnostic challenges in both pediatric and adult infections. However, distinguishing contamination from true bacteremia remains a challenge, as CoNS may act as opportunistic pathogens in neonates, immunocompromised patients, or those with indwelling devices [1-4]. True infection in immunocompetent children is extremely rare and often debated. Our patient presented with persistent metabolic acidosis during a febrile lower respiratory tract infection, but with stable hemodynamics and no organ dysfunction or ongoing bacteremia, highlighting this diagnostic dilemma.

In this case, metabolic acidosis, characterized by low bicarbonate, mild lactate elevation, and ketonuria, likely resulted from mild dehydration and catabolic stress, rather than infection-related hypoperfusion, as there was no evidence of shock or renal compromise [5]. In the literature, metabolic acidosis in *S. hominis* bacteremia occurs as a non-specific feature of severe sepsis or multi-organ failure, not as a direct bacterial effect [6,7]. Reported pediatric cases nearly always involve significant risk factors [8-10]. Our case is unique in describing a healthy child with only transient acidosis and no signs of severe infection, underlining the rarity of true *S. hominis* bacteremia in this group.

When *S. hominis* was isolated from a single blood culture, a comprehensive assessment was triggered. CoNS are the most common cause of false-positive blood cultures in children, with contamination rates up to 40% [2,5,10,11]. Criteria that favor true infection include multiple positive cultures, persistent fever, signs of sepsis, or host risk factors [2,4,10]. In our case, only the initial culture was positive, symptoms quickly resolved, and there were no risk factors, supporting the diagnosis of contamination and justifying antibiotic de-escalation.

True *S. hominis* bacteremia has been reported, primarily in neonates with central lines, oncology patients, or those with severe comorbidities, and is rare [4,8-10]. These cases often involve persistent bacteremia and systemic inflammation, and metabolic acidosis arises mainly in the context of severe sepsis. By contrast, our patient had no such risk factors and recovered rapidly with supportive care.

CoNS, including *S. hominis*, can trigger host immune responses via recognition of their cell wall components (peptidoglycan, lipoteichoic acids) by Toll-like receptor 2, leading to the release of cytokines such as interleukin-1β, tumor necrosis factor-α, and interleukin-6 [11,12]. In genuine infections, this immune activation can contribute to systemic inflammation, endothelial dysfunction, and alterations in tissue perfusion, all of which may result in metabolic derangements including lactic acidosis. However, *S. hominis* is considered less virulent than *S. aureus* and possesses limited exotoxin production and reduced capacity for rapid tissue invasion [6,7]. Chronic or biofilm-associated infections by CoNS, while significant in specific populations, were not relevant to our patient, who lacked risk factors and responded rapidly to supportive management.

Clinically, this case illustrates the importance of careful interpretation of single CoNS-positive cultures in otherwise healthy pediatric and adult patients. Overuse of antibiotics can be avoided by integrating clinical findings, repeat cultures, and risk assessment. Common causes of metabolic acidosis in children, such as dehydration and increased catabolism, should be prioritized in differential diagnosis, and multidisciplinary input is valuable when ambiguity remains.

## Conclusions

The case highlights the diagnostic and therapeutic challenges posed by the isolation of *S. hominis* in blood cultures from pediatric patients with metabolic acidosis. In immunocompetent children, a single *S. hominis*-positive blood culture in the absence of risk factors, persistent symptoms, or positive repeat cultures should raise strong suspicion of contamination rather than true bacteremia. Nonetheless, the temporal association with metabolic acidosis in our case underscores the diagnostic challenges posed by CoNS in pediatrics. Careful interpretation and clinical correlation are essential to avoid both overtreatment and missed diagnoses. Further studies are warranted to better define the clinical significance and metabolic impact of CoNS in otherwise healthy children.
